# Other People’s Money: The Role of Reciprocity and Social Uncertainty in Decisions for Others

**DOI:** 10.1037/npe0000063

**Published:** 2017

**Authors:** Ivo Vlaev, Brian Wallace, Nicholas Wright, Antoinette Nicolle, Paul Dolan, Raymond Dolan

**Affiliations:** 1 Warwick Business School, University of Warwick; 2 Department of Economics, University College London; 3 Institute for Conflict, Cooperation and Security, University of Birmingham; 4 School of Psychology, University of Nottingham; 5 Department of Psychological and Behavioural Science, London School of Economics; 6 Max Planck Centre for Computational Psychiatry and Ageing and Wellcome Trust Centre for Neuroimaging, University College London

**Keywords:** decision-making, risk preferences, time preferences, self–other differences, reciprocity

## Abstract

Many important decisions are taken not by the person who will ultimately gain or lose from the outcome, but on their behalf, by somebody else. We examined economic decision-making about risk and time in situations in which deciders chose for others who also chose for them. We propose that this unique setting, which has not been studied before, elicits perception of reciprocity that prompts a unique bias in preferences. We found that decision-makers are less patient (more discounting), and more risk averse for losses than gains, with other peoples’ money, especially when their choices for others are more uncertain. Those results were derived by exploiting a computational modeling framework that has been shown to account for the underlying psychological and neural decision processes. We propose a novel theoretical mechanism—precautionary preferences under social uncertainty, which explains the findings. Implications for future research and alternative models are also discussed.

You can spend your own money on yourself. When you do that, why then you really watch out what you’re doing, and you try to get the most for your money. . . . Finally, I can spend somebody else’s money on somebody else. And if I spend somebody else’s money on somebody else, I’m not concerned about how much it is, and I’m not concerned about what I get. And that’s government. And that’s close to 40% of our national income.

—Milton Friedman, *Fox News* interview (May 2004)

Decisions on behalf of others are ubiquitous across much human social and economic activity (Stiglitz, 1987), from doctors taking decisions on behalf of patients to city traders deciding on behalf of investors (Wonderling, Gruen, & Black, 2005). For example, investors delegate to traders the responsibility to manage their money, which the former cannot continuously monitor and control. This is the case for most contracts written in a world of information asymmetry, uncertainty, and risk. It is crucial, therefore, to understand how people actually make decisions on behalf of others and how this differs from when they make decisions on their own behalf. Taking a doctor as an example, subject to resource constraints, she might act to maximize a patient’s health (Culyer, 1989; Williams, 1988), maximize the economic utility of the patient by minimizing the financial cost (Evans, 1984), or maximize social welfare by minimizing the cost to society (Mooney, 1994).

A rich plethora of studies have reported how people decide on behalf of others. Examples include studies using nonmonetary decision outcomes (Beisswanger, Stone, Hupp, & Allgaier, 2003; Garcia-Retamero & Galesic, 2012; Kray, 2000; Kray & Gonzalez, 1999; Polman, 2012a; Stone, Choi, de Bruin, & Mandel, 2013; Wray & Stone, 2005; Zikmund-Fisher, Sarr, Fagerlin, & Ubel, 2006), hypothetical monetary outcomes (Bartels & Rips, 2010; Borresen, 1987; Hsee & Weber, 1997; Takahashi, 2007; Ziegler & Tunney, 2012), between-subjects designs that do not directly compare the within-person decisions made for oneself with the same decisions made for somebody else (Fernandez-Duque & Wifall, 2007; Pronin, Olivola, & Kennedy, 2008), outcomes that are only in the domain of risky gains without the possibility for losses (Stone, Yates, & Caruthers, 2002; Teger & Kogan, 1975), and within-subject designs that compare outcomes in the domain of both risky gains and risky losses (Cvetkovich, 1972; Polman, 2012a).

In a review of the literature of risk taking for others, Polman (2012b) stressed that

much of the research on choosing-for-others deals with risk preferences, with some research reporting that decisions tend to be more risky when made on behalf of others (Beisswanger et al., 2003; Stone et al., 2002; Ubel, Angott, & Zikmund-Fisher, 2011; Wray & Stone, 2005) and other research reporting that decisions tend to be less risky when made on behalf of others (McCauley, Kogan, & Teger, 1971; Teger & Kogan, 1975; Wallach, Kogan, & Bem, 1964; Zaleska & Kogan, 1971). (p. 142)

Given this doubt about whether choices for others are more or less risky as compared with choices for the self, Polman (2012b) answered a different question: whether choices that people make for others are less loss-averse. Andersson, Holm, Tyran, and Wengström (2016) found that deciding for others reduces loss aversion (less risk taking for self than others when gains and losses are possible). In intertemporal choices, the evidence is also mixed, with some research reporting that deciders are more patient (discount less) when deciding for others (Pronin et al., 2008; Ziegler & Tunney, 2012), and other research reporting that decisions tend to be more impatient (discount more) when deciding for others (Bartels & Rips, 2010; Takahashi, 2007). In summary, the mixed evidence for surrogate decisions clearly suggests that situational or contextual factors may be mediating those effects.

A characteristic feature of the design of all previous studies is that decision-makers are asked to make choices either for self, others, or both self and others. In contrast to this work, *we uniquely study the kind of situations in which decision-makers make decisions for others who also make decisions for them*. This is an important topic to understand, as many in important relationships make decisions for each other. In any team situation, individuals are making decisions for others as well as on behalf of themselves. This also includes people in close relationships, close friends, and married couples (e.g., spouses make decisions for each other frequently). Also, many situations in which strangers are brought together to form a team with others to perform a task contains such reciprocal decision-making (e.g., in the military, in business, and even some tasks in the TV program *The Apprentice*). Individuals working in large organizations may also make such, often anonymous, decisions for each other.

Because in reality there are many confounding factors that could explain preferences, such as memory of previous interactions, our design strips the decision task down to an anonymous interaction that preserves only the “reciprocity” element under a set of neutral baseline conditions: confidentiality of the decisions and lack of opportunity for retaliation. Thus, the “you choose for me and I choose for you” paradigm, in which the “other” subject is just chosen at random, should make only “reciprocity” more salient, so we can study its unique effect on preferences in such situations. As it turns out, none of the previous studies in the literature have employed this setting.

## Uncertainty Moderates Decisions for Others

Social interactions are plagued with uncertainty because actors can never truly know what it is like to see or experience something from someone else’s point of view (Harsanyi, 1977; Nagel, 1974). Instead, people must rely on their best estimates of others’ beliefs and preferences to guide social decision-making (Yoshida, Dolan, & Friston, 2008) and choose carefully when outcomes affect the fate of others. Therefore, a likely moderator of decisions for others is *uncertainty* about others’ preferences and goals. Conversely, if we are certain about what others want, regardless of how different from our own values, we are likely to implement those preferences in our choices for them (see Nicolle et al., 2012 for evidence that decisions for others are attuned to others’ preferences, and draw on brain mechanisms used when decisions are made for the self, if the actors have the opportunity to learn what the other person would decide in a similar situation).

## Computational Modeling of Decisions for Others

Computational modeling of the underlying decision processes is informative in this task because it is not clear whether decisions for others reveal preferences or some non-preference-based decision strategies (see DellaVigna, 2009). Simply observing behavioral choices does not tell us what the underlying mechanisms of such decision-making may be. Specifically, according to popular economic and behavioral theories (DellaVigna, 2009), there are three stages through which decisions are made: (a) start with underlying preference (desires, “wants,” and “needs”); (b) then form beliefs about likelihood/probability of different outcomes; (c) and use a decision-making rule to assess the available information and make a choice that satisfies the preferences, given the beliefs. Psychological factors can affect each step. For example, preferences are affected by the desire to avoid unpleasant emotions such as regret (Loomes & Sugden, 1982). Moreover, urges for immediate gratification create self-control problems and challenge the assumption that the discount factor is time-consistent (Laibson, 1997), and reference points challenge the assumption that decision utility depends on total lifetime wealth (Kahneman & Tversky, 1979). Beliefs are often formed following intuitions incapable of assessing actual risk, which lead to overconfidence about one’s abilities (Tversky & Kahneman, 1992). Different decision rules or heuristics are adopted to simplify decisions under the constraint that rationality is *bounded* (Simon, 1997).

Computational neuroscience and economics have offered a range of approaches to model such phenomena during those three stages of the decision-making process (Glimcher, Camerer, Fehr, & Poldrack, 2009; Rangel, Camerer, & Montague, 2008). The parameters of these models have even been found to correlate with neural activity in specific brain structures, which suggest that those models might provide a mechanistic level of explanation of overt decision behaviors (see Glimcher et al., 2009). We employ such computational models to test the specific hypotheses about how individuals make financial decisions involving risk and time for self and others. Such models are designed to reveal whether specific “preferences” and “decision rules” account for observed behavioral choices.^1^

In terms of this decision model, the uncertainty mechanism makes a prediction concerning the “decision rule” that translates (time and risk) preferences into choices. Uncertainty in decision-making is reflected in choice noisiness, or, more formally, the reliability with which decision values are translated into choices (De Martino, Fleming, Garrett, & Dolan, 2013). The uncertainty principle makes two crucial predictions. First, it implies more noisy choices for others than for self in general. Second, if uncertainty moderates decisions for others, then preferences should be different between deciders with more and less noisy choices for others respectively. We tested those two predictions by assessing the noise in participants’ choices by fitting a stochastic decision rule with a noise parameter and estimating the parameter for decisions for self and others, respectively.

## Experiment 1: Intertemporal Choice for Self and Others

To examine preferences and decision rules for self and others, we designed an intertemporal choice task, which asked participants to make choices between larger rewards delivered *later*, and smaller rewards delivered *sooner*, sometimes for themselves and other times on behalf of an anonymous other participant. It is known that different individuals display significant variability in their preferences, with relatively low discounters preferring to wait for a *later* higher reward option and relatively high discounters preferring the more immediate smaller reward (Kable & Glimcher, 2007; Pine et al., 2009). This behavioral variability can be quantified by estimating an individual’s *discount rate*, a parameter which quantifies an individual’s disposition to discount the value of delayed relative to more immediate rewards. We were also interested in model parameters that could distinguish individuals both in their valuations and choice preferences. In previous studies of value comparison, ventromedial prefrontal cortex activity has been found to correlate with the subjective value of a chosen option or with the value difference between chosen and unchosen options (Basten, Biele, Heekeren, & Fiebach, 2010; Fitzgerald, Seymour, & Dolan, 2009) both when deciding for oneself and for others (Nicolle et al., 2012).

### Method

#### Participants

Forty-five participants (23 male and 22 female) were randomly allocated to two groups of 22 and 23 subjects, respectively. All subjects provided informed consent and the study was covered by approval granted by University College London Research Ethics Committee. All subjects were students at London universities (the vast majority were undergraduates and from University College London).

#### Design

Subjects were invited to the laboratory and seated in separate cubicles to ensure privacy in decision-making. To reinforce the social nature of the task, they were given written instructions (see Appendix A), which emphasized that their payoff may be determined by another subject’s choices, in which case the other subject’s payoff would be determined by their own choices. The study was conducted using the z-Tree software (Fischbacher, 2007).

The study consisted of 240 trials in which, in each trial, a subject chose between two amounts to be received at different dates in the future for a beneficiary. The main treatment variable was a within-subject variation in the beneficiary of the decisions, which could be either “me” (i.e., the subject themselves), “other (female)” (i.e., another subject in the lab that was female), and “other (male).” In addition to being displayed on the screen, the background of the screen was color coded depending on the beneficiary: black for self, light blue for another male, and pink for another female.

**Figure 1 fig1:**
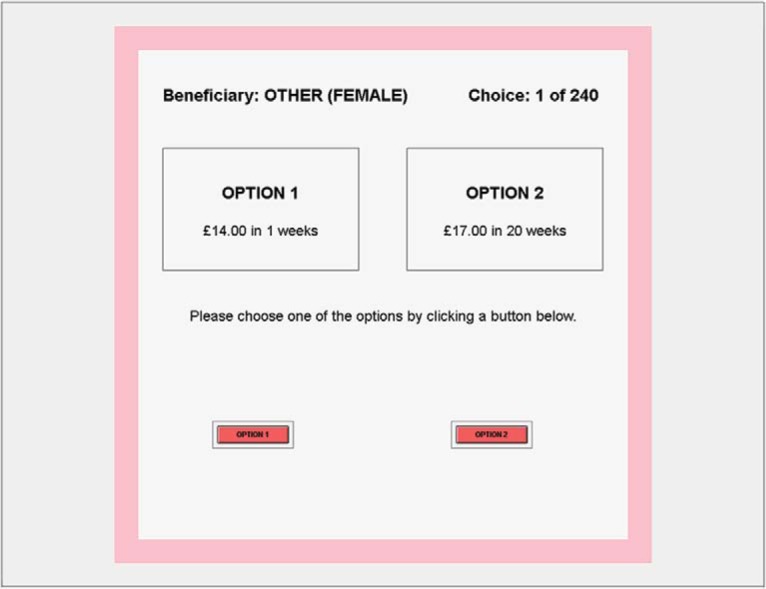
An example trial screenshot for the delegated intertemporal choice task.

In each trial, the subject was shown a screen with the following information: the beneficiary of the trial (i.e., if that trial is chosen, this determines who receives the payoff), the trial number (from 1 to 240), and two payment options (which are framed as “Option i: £*x* in *y* weeks”) together with two buttons. Subjects could press only one button, corresponding to their chosen option. To prevent accidental double clicks and to encourage subjects to think, the buttons did not appear until a second after the options were displayed. The options always had a higher amount of money at a *later* date (e.g., £17 in 20 weeks) in one option (the *later* option) and a lower amount of money at a *sooner* date (e.g., £14 in 1 week) in the other option (the *sooner* option), but which option was labeled Option 1 (and presented on the left of the screen) and which was labeled Option 2 (and presented on the right) was chosen at random; this enabled us to identify subjects that may have been consistent in their behavior only because they clicked the same button each time. A screenshot of a specific trial is shown in Figure 1.[Fig-anchor fig1]

The *sooner* option payment date was always either 0, 1, or 2 weeks (0 weeks being the day of the experiment) and the *later* date varied between 1 and 25 weeks; the *sooner* payment amount varied between zero and £24 and the *later* payment amount varied between £6 and £49. A number of attention checks were included in the parameter choices, that is, options in which the payment size was the same but the dates were different, options in which the dates were the same but the payment size was different, and options in which the payment size was zero.

There were 80 trials containing different possible combinations of magnitudes and delays reflecting a wide range of implied discount factors (for more information how the options were generated see Nicolle et al., 2012), and subjects faced the entire set once for each beneficiary. Trials were split up into blocks of 40 choices for the same beneficiary (all participants performed all three beneficiary conditions); the blocks were ordered at random as were the different trials within a block. There was a 30-s time between blocks for rest.

Although subjects could make decisions at their own pace (there was no time limit—hence, also no incentive to rush decisions), a session could not be completed until all subjects had completed their decision-making (as payoffs may depend on other subject’s decisions). After all subjects had completed all of their choices, subjects were randomly assigned to be paid based either on their own choice or the choice of another subject, and one choice was randomly selected for each subject to be paid.

In total, each session lasted about 65 min, including reading instructions and dealing with payment administration. To completely isolate the effect of time discounting on decision-making, there was no show up fee in this experiment, and subjects earned on average £25.64. The money was credited into the subjects’ bank account or via PayPal at the time specified in the payoff-relevant choice (e.g., if the payoff-relevant choice was to be paid £17 in 20 weeks, they were paid £17 exactly 20 weeks after the experiment).

#### Behavioral modeling

A function that describes the pattern of discounting can be estimated by observing choices between delayed outcomes. Economic theories of rational behavior posit that goods ought to be discounted exponentially with delay (Samuelson, 1937). Formally, an outcome that has value or utility *M* if received immediately (*T* = 0) is worth γ^*T*^ · *M* if delayed *T* periods into the future. The present-time utility *U* of receiving *M* at time *T* is thus given by

$$U\;=\;{\text{γ}}^{T}\cdot M.$$ 1

Here, the discount rate, γ, represents the constant proportional decrease in value with each added time period of delay. Contrary to predictions of exponential discounting, a large body of evidence with both human and animal subjects has demonstrated that discount rates appear to decrease with increasing delays (Green & Myerson, 2004; Rubinstein, 2003). Again, each participant’s unique discount rate was estimated by fitting a discount function to their choices, whereby the discounted utility/value of an option (*U*) varies hyperbolically as a function of reward or payoff magnitude (*M*) and delay (*T*):

$$U\;=\;\frac{M}{1+\text{γ}T}.$$ 2

A proposed alternative is the “quasihyperbolic” approximation to hyperbolic discounting (Laibson, 1997), which is formalized as exponential discounting, with an additional preference for immediate rewards (present bias), expressed as a second discount factor, δ, and applied to all time periods except the first:

$$U\;=\;M_{T0}+\text{δ}({\text{γ}}^{T}\cdot M_{T}),$$ 3

where *M*_*T0*_ is the immediate payoff, *M*_*T*_ is the payoff in period *T*, δ is an individual’s present bias parameter, and γ is the individual’s long-run discount factor. When δ = 1, individuals are not present biased and the quasihyperbolic model reduces to standard exponential discounting. Such present-biased time preferences may be the result of the interplay between two separate decision-making systems: the affective system that values immediate gratification and the deliberative system that makes long-run plans and displays higher discount factors (McClure, Laibson, Loewenstein, & Cohen, 2004).

Given a subjective (i.e., temporally discounted) value associated with each of the two (*sooner* vs. *later*) options in the pair, the associated probability of making each choice is estimated with a softmax (logistic) decision rule, in which β > 0 determines the randomness of the decision—higher numbers indicate more deterministic, less random choice (in our simulations, β was constrained between 0 and 10):

$$P(Later)\;=\;\frac{1}{1+e^{-\text{β}\;(U(Later)-U(Sooner))}}.$$ 4

This is a standard stochastic decision rule that calculates the probability of taking one of two actions according to their relative subjective values (see Glimcher et al., 2009). This model also describes neural signals in medial prefrontal cortex, which compute the choice preferences of another individual as well as oneself—both in terms of subjective valuations of options and in terms of choices (see Nicolle et al., 2012).

Critically, it is necessary to optimize the choice pairs such that they give the most efficient estimate of potential subjects’ discount rates. To optimize these choices, we generated a random 80 choice pairs, each comprising one smaller, *sooner* reward and one larger, more delayed reward, but with the magnitudes and delays varying across the pairs. We then computed the decisions predicted to be made by simulated subjects with discount rates ranging from 0 to 1. When plotted those simulated discount rates against the predicted number of choices of the delayed option, the closer this graph is to the diagonal, the better different discount rates are reflected in different subject choices, and, therefore, the lower the error introduced by the model estimation process (Nicolle et al., 2012). We generated 10,000 such choice sets and chose the set whose curve was closest to the diagonal (in terms of enclosed area). In this choice set, magnitudes ranged between £0 and £24 for *sooner* options, with their delays ranging from 0 to 2 weeks. For the more delayed options of each pair, the magnitudes ranged from £6 to £49, and the delays from 1 to 25 weeks, as these provided the lowest correlations in self and other choices and values.

The three choice models (Equations 1–3) were separately fit to participants’ choices in the prescreen questionnaire, optimizing their free parameters (γ, δ, and β) to maximize the likelihood of the choices (Equation 4), given the parameters. This was realized through standard MATLAB functions used to compute maximum likelihood estimation. Model comparison was performed at the group level, by summation of individual log likelihoods. Selection between models proceeded using the Bayesian information criterion (BIC; Schwarz, 1978), in which *L* is the maximized group-level log likelihood, *k* is the number of free parameters in the model, and *n* is the number of independent observations:

BIC=–2L+2kln(n). 5

For BIC, Kass and Raftery (1995) propose the following taxonomy of what constitutes a substantial difference in criterion values: 0–2 = weak, 2–6 = positive, 6–10 = strong, and >10 = very strong. We used BIC to compare between three versions of the model.

Finally, after selecting the winning model, we included in the analysis only the participants for whom the model performed above certain minimum goodness-of-fit measure. We applied McFadden’s (1979) pseudo-*R*^2^ to measure the improvement from null model to fitted model:

R2=1−lnL^(MFull)lnL^(M0), 6

where *M*_*Full*_ is the model with predictors, *M*_*0*_ is the model without predictors that assumes random choice, and L^ is the estimated likelihood. The likelihood for the null model is defined as follows:

L^(M0)=k1N, 7

where *N* is the number of choice options (each choice is equally likely, so with two options 1/*N* = 0.5) and *k* is the number of observations or choices. Note that the theoretical range is 0 < McFadden pseudo-*R*^2^ < 1, but as a rule of thumb, the model has an excellent fit when 0.20 < McFadden pseudo-*R*^2^ < 0.40 (McFadden, 1979).^2^

### Results

The computational modeling reveals important variance in the data, because it allows us to separate the effects preferences and choice uncertainty. The impact of delay depends on the participant’s preferences measured by their unique discount rate (γ), which was constrained to fall between 0 and 1. In our task, this was a reasonable constraint, as a subject with a discount rate of 1 would always choose the smaller *sooner* option on trials in which the delay and magnitude favored opposite choices. The hyperbolic discount model produced the best model fit (BIC = 6,273) compared with the exponential model (BIC = 6,283) and the quasihyperbolic model (BIC = 6,854). (Note that smaller numbers reflect a better fit.) With our winning hyperbolic model, replacing the separate parameters for Other decisions for males and females (β_male_ and β_female_; γ_male_ and γ_female_) with the single gender-independent free parameter (β_other_ and γ_other_) resulted in even better BIC performance: 5,956 versus 6,273, respectively. Here, we present the statistical analysis of the parameters of this best-fitting self-other hyperbolic model (although similarly significant results are obtained when analyzing the parameters of the model with separate male and female parameters).

We excluded from the analysis four participants (two male and two female) for whom the model showed unsatisfactory McFadden pseudo-*R*^2^ goodness-of-fit measure (mean *R*^2^ = .12), thus analyzing the data from the 41 remaining participants (mean *R*^2^ = .72). Table 1 presents the modeling results and the behavioral choices in each condition according to the beneficiary (self and other). The discounting parameters (γ) from this winning model and the simpler metric (proportion delayed choices) were highly correlated for self (*r* = −0.81) and other (*r* = −0.83). We analyzed the distribution of the inferred individual time discounting parameters for self and other. The differences between the self and other conditions were examined with nonparametric statistical analysis because this variable was not normally distributed according to the Kolmogorov–Smirnov test of normality for self (*D* = 0.33, *p* < .001) and other (*D* = 0.31, *p* < .001). The Wilcoxon signed-ranks test revealed that the discounting parameter (γ) was significantly higher in other beneficiary condition compared with self, *Z* = 2.78, *p* = .006. The behavioral data from those participants revealed the same pattern. The proportion of delayed choices was significantly higher in the self beneficiary condition as compared with other, *Z* = 3.31, *p* = .001, *t*(40) = 3.02, *p* = .004, using paired-samples *t* test.^3^ The individual level analysis revealed that for 27 of the participants the time preference estimate γ was smaller when making decisions for oneself than for the other (*M* = 0.06, *SD* = 0.18), whereas 14 participants exhibited the opposite pattern—the time preference estimate was larger when making decisions for oneself (*M* = 0.01, *SD* = 0.03). Note, however, that the magnitude of the difference is negligible when discounting for self is larger (i.e., those individuals could be considered as having equal discounting for self and other).[Table-anchor tbl1]

We also analyzed how the choice randomness parameter (β) varied across the two conditions, which tested the key prediction that the uncertainty in decision-making is reflected in choice noisiness, or more formally the fidelity with which decision values are translated into choices (De Martino et al., 2013). Recall that the uncertainty principle predicts more noisy choices for others than for self. Also, if uncertainty moderates decisions for others, then those preferences should be different between deciders with more or less noisy choices for others respectively. We used nonparametric statistical analysis, because this variable was not normally distributed according to the Kolmogorov–Smirnov test of normality for self (*D* = 0.34, *p* < .001) and other (*D* = 0.44, *p* < .001). The Wilcoxon signed-ranks test revealed that the average β across participants was significantly higher when deciding for self beneficiary compared with other, *Z* = 3.52, *p* < .001 (i.e., decisions were less deterministic, or more random, for others). The next step in the analysis was to determine whether the difference between β for other moderates the self–other differences in discounting. We did a median split of the βs for other, which divided the sample into high and low uncertainty group depending on whether they were below or above the median β, respectively. The between-subjects tests revealed that discounting was significantly different between those groups—more discounting for lower β, that is, when the participants were more uncertain/random about the preferences of other. Specifically, the Wilcoxon signed-ranks test revealed that the discounting parameter (γ) was significantly higher for other beneficiary in the high (*M* = 0.12, *SE* = .05; *Mdn* = 0.06) as compared with the low (*M* = 0.09, *SE* = .05; *Mdn* = 0.02) uncertainty group respectively, *Z* = 2.44, *p* = .015. The self–other differences in discounting was also higher in the high (*M* = 0.07, *SE* = .05; *Mdn* = 0.02) as compared with the low (*M* = 0.01, *SE* = .01; *Mdn* = 0.00) uncertainty group, *Z* = 2.53, *p* = .012. The behavioral data from those participants revealed the same pattern. The proportion of delayed choices for other was significantly smaller in the high (*M* = 0.68, *SE* = .04; *Mdn* = 0.69) compared with the low (*M* = 0.82, *SE* = .05; *Mdn* = 0.90) uncertainty group, *Z* = 2.78, *p* = .005. Similarly, the self–other difference in proportions of delayed choices was higher in the high (*M* = 0.09, *SE* = .03; *Mdn* = 0.07) as compared with the low (*M* = 0.01, *SE* = .01; *Mdn* = 0.00) uncertainty group, *Z* = 2.77, *p* = .006. Note that the differences between the high and low uncertainty groups were examined with nonparametric statistical analysis because all variables were not normally distributed according to the Kolmogorov–Smirnov test of normality for other γ (*D* = 0.31, *p* < .001), self–other γ (*D* = 0.34, *p* < .001), other proportions (*D* = 0.15, *p* = .019), and self–other proportions (*D* = 0.20, *p* < .001).

### Discussion

The behavioral results and the modeling parameters for time discounting showed that individuals are more impulsive (less patient) with other peoples’ money. Our modeling results also revealed that choices are more random when making time–money trade-offs for others, which indicates uncertainty about their preferences. We also observed more impatient decisions for others in decision-makers with noisier choices for the others, which supports the prediction that uncertainty about others’ preferences moderates decision-making. Thus, the study revealed unique biases in both the preferences and in the decision rules that individuals employ when making a choice that satisfies their time preferences (in line with the decision framework proposed at the beginning, DellaVigna, 2009).

## Experiment 2: Risky Choice for Self and Others

We also investigated whether decision-making under risk is different when people use their own money rather than somebody else’s money. We did not test for differences in behavior for male and female beneficiaries in this study for two reasons. First, because this hypothesis has already been established in risky choice for self and others (Cvetkovich, 1972; Stone & Allgaier, 2008; Stone et al., 2002), and second, because removing the gender factor from our design provided enhanced power for assessing the effect of decision (gain/loss) valence.

### Method

#### Participants

Fifty individuals participated in the study; the study was conducted in three groups of 18, 18, and 16 subjects (33 females and 17 males). All subjects had previously provided informed consent, and the study was covered by approval granted to the Department of Economics from the University College London Research Ethics Committee. All subjects were students at London universities (the vast majority were undergraduates and from University College London).

#### Design

Subjects were invited to the laboratory and seated in separate cubicles to ensure privacy in decision-making. To reinforce the social nature of the task, they were again given written instructions (see Appendix B), which emphasized that their payoff would be partly determined by another subject’s choices, and that their “partner’s” payoff would depend partly on their payoffs. The study was conducted using the z-Tree software (Fischbacher, 2007).

**Figure 2 fig2:**
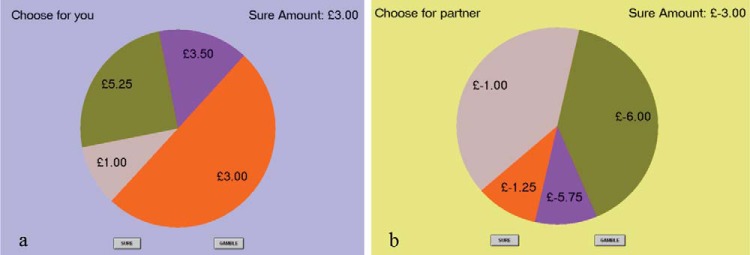
Structure of the risky decision task on two separate trials. (a) An example of a gain trial choice for self. (b) An example of a loss trial choice for other.

Screenshots of two trials are shown in Figure 2—the task was adapted from Wright et al. (2012). In each trial, participants needed to choose between a “gamble” option and a “sure” option. First, the participants saw a blank screen with fixation cross; second, they were shown the pie chart with four sectors for the gamble option and the value of the sure option. After the pie chart had been displayed for 4 s, two buttons appeared, one of which they pressed to register their choice.[Fig-anchor fig2]

If the “gamble” option is selected, subjects win one of the four specified amounts with a probability given by the corresponding area of the pie chart. If the “sure” option is chosen, subjects win the specified “sure amount” for certain. The colors (chosen from a set of neutral colors) and the order and orientation of the pie sectors are determined at random. Wright et al. (2012) showed that the degree of risk in those lotteries positively correlated with neural activity in posterior parietal cortex, a region strongly associated with risk, and the manipulation of “valence” was expressed in greater activity for gains than losses in value-related areas of orbitofrontal cortex and bilateral striatum.

The study investigated two main factors. The first factor was the effect of beneficiary, whereby subjects played half the trials on behalf of themselves and half on behalf of another subject in the laboratory. The beneficiary was displayed on the screen in each individual trial. The second factor was the effect of winning versus losing money in a trial—the so-called valence effect. Half the trials were in the gain domain, in which the gamble amounts and sure amount were positive, and the other half were in the loss domain, in which they were negative. This provided a 2 × 2 factorial design in this basic design with within-subject factors for Valence (gain or loss) and Beneficiary (self or other). For each subject, the first half of the session was played for one beneficiary and the second half for the other beneficiary; within each half, gain and loss trials were played in a random order. The color of the background screen was specific to the beneficiary type.

There were 49 distinct gamble options, and they were played four times each by each subject (once for each combination or gain/loss and self/other) for a total of 196 decisions per subject. The sure option was always a gain or loss of £3 and the gamble options ranged from £0 to £6. Thus, across the set of 49 gain trials, we parametrically manipulated the degree of risk in the lottery (using seven levels of variance) and orthogonally manipulated its expected value (EV; seven levels). One-half of the lotteries had an EV above the sure amount and one half below (mean EV across all 49 lotteries was equal to the sure option), which provided a simple metric of risk preference indexed as the proportion of riskier choices made (proportion risky choices: risk neutral, 0.5; risk averse, <0.5; risk seeking, >0.5). To manipulate valence, we created 49 perfectly matched loss trials by multiplying all amounts in our gain trials by −1 (see also Wright et al., 2012, for more information on how these trials were constructed). The full set of trial parameters was seen once in each cell of the design in every session, so the full set was shown four times in total—gain and loss for self and other each.

Participants’ payments comprised a £5 show up fee, plus a £12 initial endowment (allowing for any losses occurred in the loss frame trials), plus the outcome of four randomly chosen trials—two in which the subject chose on behalf of themselves (one gain and one loss frame) and two in which another subject chose on their behalf (one gain and one loss frame). The £12 covered the worst possible outcome (in which participants won £0 in the gain frame and lost £6 in the loss frame). Sessions lasted about an hour including reading instructions and processing payments and subjects earned an average of £17.

#### Behavioral modeling

In our analysis, we assessed whether the participants become noisier in their choices for the other person by fitting a choice model to choices in each of the four cells of the factorial design (gain/loss, self/other) in which the utility of the sure and gamble options are computed in each trial (using a mean-variance utility function), with the softmax (logistic) decision rule used to choose between them. Therefore, we estimated a risk parameter and noise parameter in each condition for each subject. We modeled individual choices using a binary logistic regression utility model. On each trial the subjective values, or utilities (*U*), of both options (gamble vs. sure) were computed using a utility function; then, these values were compared with generated trial-by-trial probability of accepting the lottery, using a softmax function with a free parameter β (constrained between 0 and 10), which allows for noise in action selection as follows:

$$P(Gamble)\;=\;\frac{1}{1+e^{-\text{β}\;(U(Gamble)-U(Sure))}}.$$ 8

Note that this randomness/noisiness parameter β is also shown to correlate with prefrontal neural structures (anterior frontopolar cortex) that control for exploration versus exploitation strategies in decision-making under uncertainty (Daw, O’Doherty, Seymour, Dayan, & Dolan, 2006). Also, in line with prefrontal cortex’ role as the principal region implicated in behavioral control, Daw et al. showed that activity in the orbitofrontal and ventromedial prefrontal cortices correlates with the probability assigned by the model to the action chosen on a given trial. (In the softmax model, this probability is a relative measure of the expected reward value of the chosen action.)

We then define the subjective value, or utility (*U*), of each lottery using the mean-variance model:

U(Gamble)=EV+ρVar. 9

*EV* is the expected (mean) value, *Var* is the variance of the gamble option. For a given lottery with *N* potential outcomes (*m*_*1*_, *m*_*2*_, . . . *m*_*N*_), with probabilities *p* = *p*_*1*_, *p*_*2*_, . . . *p*_*n*_, we define those statistical moments as follows:

$$EV\;=\;\sum_{n=1}^{N}m_{n}p_{n},$$ 10

$$Var\;=\;\sum_{n=1}^{N}{\left(m_{n}-EV\right)}^{2}p_{n},$$ 11

where ρ is a risk parameter (constrained between −1 and +1, reflecting the taste for risk, with zero indicating risk neutrality, a positive number risk seeking, and a negative number risk aversion), and β reflects choice noisiness. Equation 10 also represents the most basic *mean model*, and Equation 9 is known as the classic *mean–variance model* (Bossaerts, 2010; Markowitz, 1952). In the *mean*–*variance–valence* model, on each trial the subjective values, or utilities (*U*), of both options (*A* and *B*) were computed using the aforementioned utility function, where ρ = ρ_gain_ in gain trials and ρ = ρ_loss_ in loss trials. Wright et al. (2012) show that this *mean–variance–valence* model best predicts behavioral data in this task compared with the basic *expected value* model (without variance term), the classic *mean–variance model*, the *expected utility* model (Camerer, 2003), the *prospect theory* model (Kahneman & Tversky, 1979), and the *cumulative prospect theory* model (Tversky & Kahneman, 1992). This winning model enables extending ideas derived from financial economics that individuals respond to risk as measured by the variance in potential outcomes (Bossaerts, 2010; Markowitz, 1952), by incorporating valence. Also, Wright et al. (2012) demonstrated that the components of this model correlate with activity in brain regions in the parietal and temporal cortices, anterior insula, and the ventral striatum, which are known to encode risk information and decision utility.

In this model, there is no constant term, as we assume a utility of 0 represents a point of subjective indifference between sure or gamble (i.e., rejection of offer has a utility of 0). We optimized subject-specific ρ and β parameters using nonlinear optimization implemented in MATLAB for maximum likelihood estimation. In our simulations of the observed decisions for self and other, we extended the mean–variance model by replacing the parameters ρ and β with separate parameters for self-trials (ρ_self_ and β_self_) and other-trials (ρ_other_ and β_other_). Model fitting resulted in a set of maximum likelihood parameter estimates for each subject. Model comparison was performed at the group level (fixed effects), by summation of log likelihoods across participants. As in the previous study, selection between models proceeded using the BIC (Schwarz, 1978).

We used BIC to compare the following three models, which allowed us to ask whether decision behavior for self and other was influenced by risk and valence (i.e., every model was fit to all the data for each subject). First, a simple *mean model* (see Equation 10) assumed that individuals only cared about the mean value of the options. Second, we asked whether choice was also influenced by risk, using a *mean*–variance model [see Equation 9). Third, we asked whether both risk and valence influence choice, using a *mean*–variance–valence model with separate risk parameter that reflects risk preference in gain trials and loss trials, respectively.^4^ Again, after selecting the winning model, we included in the analysis only the participants for whom the model performed above the minimum McFadden’s pseudo-*R*^2^ goodness-of-fit measure.

### Results

The behavioral modeling revealed that the mean–variance–valence model produced the best model fit (BIC = 11,013) as compared with the mean–variance model (BIC = 11,225) and the mean model (BIC = 12,137).^5^ With our winning model, replacing the single free parameter in our softmax decision rule (β) with separate parameters for gain trials (β_gain_) and loss trials (β_loss_) resulted in worse BIC performance: 11,013 versus 11,388, respectively.

We included in the analysis the 40 participants for whom the model showed satisfactory McFadden’s pseudo-*R*^2^ goodness-of-fit measure (mean *R*^2^ = .35), whereas for the excluded 10 participants this measure indicated that their behavior was random and inconsistent (mean *R*^2^ = .15; McFadden, 1979). Risk-related parameters (ρ) from this winning model and the simpler metric (proportion risky choices) were highly correlated for gain and loss trials in both conditions (self-gain, *r* = .80; self-loss, *r* = .95; other-gain, *r* = 78; other-loss, *r* = .82).

Table 2 presents the behavioral and modeling results in each condition. Analyzing the data from those participants alone, differences between conditions were examined with a repeated-measures two-way (Beneficiary × Valence) univariate analysis of variance (general linear model), with the mean risk aversion parameter (ρ) as the dependent measure. There was no significant main effect of Beneficiary, *F*(1, 39) = 0.09, *p* = .765 (η^2^ = .00), indicating that those participants were not more risk averse when deciding for self (vs. other). There was still a significant main effect of Valence, *F*(1, 39) = 9.99, *p* = .003 (η^2^ = .20), which means that the inferred risk aversion parameter was significantly lower (more risk averse) for losses. The Beneficiary × Valence interaction was significant, *F*(1, 39) = 5.34, *p* = .026 (η^2^ = .12), which confirms our hypothesis that the gain–loss asymmetry in choices is bigger when deciding for others.[Table-anchor tbl2]

The behavioral data from those participants showed the same pattern. For each individual, the behavioral proportion of risky choices indexes risk preference, which was derived separately for each cell in the (Beneficiary × Domain) design: self-gain, self-loss, other-gain, other-loss. The differences between the conditions were examined with a repeated-measures two-way (Beneficiary × Domain) univariate analysis of variance (general linear model using probit link function), with the mean proportion of risky choices as the dependent measure. There was no significant main effect of Beneficiary, *F*(1, 39) = 2.60, *p* = .115 (η^2^ = .06), indicating that those participants were not more risk averse when deciding for self (vs. other). There was significant main effect of Valence, *F*(1, 39) = 6.19, *p* = .017 (η^2^ = .14), which means that the inferred risk aversion parameter was significantly lower (more risk averse) for losses. The Beneficiary × Valence interaction was significant, *F*(1, 39) = 3.68, *p* = .063 (η^2^ = .09), which confirms that the gain–loss asymmetry in choices is different between beneficiaries.

Finally, we analyzed how the choice randomness parameter (β) varied across the two conditions, because uncertainty in decision-making is reflected in choice noisiness—the fidelity with which decision values are translated into choices (De Martino et al., 2013). The choice randomness parameter (β) was significantly higher in the self beneficiary condition as compared with other, *t*(39) = 3.07, *p* = .004 (using paired-samples *t* test). This indicates that the choices for other were more uncertain (less deterministic) than for self. If uncertainty when choosing for others moderates risky decision-making, then the valence effect might be different between deciders with more noisy choices for others compared with deciders with less noisy choices for others. Testing this prediction involves determining whether the degree of randomness (uncertainty) for other explains the magnitude of the valence effect when deciding for others. Our analysis supported this prediction. Again, we did a median split of the βs for other, which divided the sample into high and low uncertainty groups depending on whether they were below or above the median β, respectively. The between-subjects tests revealed that the valence effect was significantly different between those groups—the difference between risk aversion for gains compared with losses was bigger when β was low, that is, when the participants were more uncertain about the preferences of other. Specifically, the Wilcoxon signed-ranks test revealed that the difference between the risk aversion parameter (ρ) for gain and loss for other was significantly bigger in the high uncertainty group (*M* = 0.19, *SE* = .06; *Mdn* = 0.16) as compared with the low uncertainty group (*M* = 0.06, *SE* = .03; *Mdn* = 0.05), *Z* = 2.30, *p* = .021. The differences were examined with nonparametric statistical analysis, because this variable was not normally distributed according to the Kolmogorov–Smirnov test of normality (*D* = 0.17, *p* = .006). The behavioral data from those participants revealed the same pattern. The difference between the proportion of risky choices in the gain and loss domain for other was significantly bigger in the high uncertainty group (*M* = 0.14, *SE* = .04; *Mdn* = 0.13) as compared with low uncertainty group (*M* = 0.03, *SE* = .03; *Mdn* = 0.05), *t*(38) = 2.05, *p* = .047. The difference was examined with standard parametric statistical *t* test, because this variable was normally distributed (*D* = 0.07, *p* = .200).

### Discussion

The novel findings from this experiment concern differences in risk preferences and decision rules that individuals employ when making choices for others compared with choices for self in reciprocal situations. We found bigger gain–loss asymmetry in risk preferences when deciding for others as compared with oneself (i.e., more risk-averse choices in the domain of losses, relative to gains, when choosing on behalf of others). This counterintuitive result was revealed only thanks to the use of mechanistic modeling that accounts for the underlying choice processes. We also found that choices are more random when deciding for others, irrespective of the underlying risk attitude, which supports the prediction about the role of uncertainty in decision-making for others. This was also revealed by the computational model employed in the analysis. We also observed larger valence effect for others in decision-makers with noisier choices for others, which supports the prediction that uncertainty about others’ preferences moderates risky decisions. This specific result could also have important implications for understanding how such decisions are made in the real world.

Our results replicate the greater gambling for gains than losses, which was repeatedly shown previously in this task (Wolf, Wright, Kilford, Dolan, & Blakemore, 2013; Wright et al., 2012; Wright, Morris, Guitart-Masip, & Dolan, 2013; Wright, Symmonds, Morris, & Dolan, 2013). Wright et al. explained these findings within a biologically grounded, process-based account of choice that progresses from option evaluation to action selection. At a mechanistic level, in the brain, it has been argued that there are a number of interacting valuation systems that together determine choice, including a separate the Pavlovian system for automatic approach–avoidance responses and a goal-directed system for reflective planning (Rangel et al., 2008). This model predicts that in the choice process, risk and valence (gain/loss) independently engage automatic approach–avoidance mechanisms. In simple instrumental tasks approach–avoidance mechanisms underlie important valence effects, evident in a close coupling between punishment and no-go (avoid) responses, and between reward and go (approach) responses (Dickinson & Balleine, 2002; Guitart-Masip et al., 2012). Wright et al. (2013) showed valence perturbs an individual’s choices independently of the impact of risk, and causally implicate approach–avoidance processes as important in shaping risky choice (individuals exhibit base level of risk taking consistent across time and context, but valence perturbs choices around that base level). Depending on context, this mechanism can produce either risk aversion for gains and risk seeking for losses or vice versa. Wright et al. showed that individuals chose a riskier option less often with losses when the instrumental requirement was to approach (select) as opposed to avoid (accept/reject) it. The observation that stimuli signaling loss induce avoidance is consistent with our design, in which the number of potential losses in the gamble (that does not contain £0 loss outcome) is more salient than the single loss option, thus triggering avoidance of the gamble. This leads to more risk aversion for loss-gambles than for gain-gambles (in which the number of potential gains triggers approach tendencies instead). In summary, those studies (including ours) illustrate the variety of intricate ways in which context determines how humans respond to aversive stimuli.

## General Discussion

The behavioral results and the computational modeling reveal that individuals are less patient and more risk averse for losses than for gains with other peoples’ money. These unique findings are contingent on the kind of situations we are studying—ones in which decision-makers make decisions for others who also make decisions for them. This setting, unique among previous research on self–other decision-making, is likely to elicit perception of reciprocity. Next, we offer possible explanations of the observed behaviors.

### Reciprocity Triggers Precautionary Decisions

The perceived reciprocity in such situations might affect decision-making in a specific way. In particular, reciprocity could motivate people to minimize others’ costs, relative to their own cost. In policy making, the *precautionary principle* prohibits actions that carry a risk of causing harm and imposes that decision-makers should prove that actions are harmless (Sunstein, 2005). This moral principle, or attitude, is consistent with observations that people dislike causing bad outcomes, especially outcomes that affect others (Cushman, Gray, Gaffey, & Mendes, 2012; Ritov & Baron, 1990; Tetlock & Boettger, 1994), which also reflects a widespread social norm that prohibits harming others and results in punishment when violated (Fehr & Fischbacher, 2004; Henrich et al., 2010). In decision-theoretic (cost-benefit) terms, every “harm” (such as pain, monetary loss, and time delay) is conceptualized as a specific type of “cost,” which should elicit precautionary decision making for others. For specific harms, such as pain, such “moral sentiment” (see Smith, 1759) even leads to a disposition to overvalue others’ suffering, relative to one’s own, which is elegantly demonstrated by Crockett, Kurth-Nelson, Siegel, Dayan, and Dolan (2014), who found that majority of people selflessly sacrificed more money to prevent a stranger’s pain than their own pain (i.e., when participants were responsible for others’ pain, most of them evaluated the cost of that pain as higher than their own). In this respect, Crockett et al. proposed that in decisions about losses, if decision-makers assume that the recipient’s mapping from a given level of cost to subjective unpleasantness is nonlinear, then this uncertainty could induce a form of risk premium in the moral costs of imposing what might be intolerable cost on another. At this stage, decision-makers prefer to avoid these moral costs by adopting a conservative decision strategy leading them to systematically err on the side of reducing others’ pain.

There are solid arguments why a reciprocity context would heighten precautionary preferences. One idea proposed by Crockett et al. (2014) is that such preferences likely evolved in a reciprocity context, that is, in real life most behavior is public and harming others will trigger punishment. So to avoid this potential punishment people err on the side of caution. Social norms that prohibit harm to others are widespread, and violation of these norms is often punished (see Buckholtz & Marois, 2012; Fehr & Fischbacher, 2004; Henrich et al., 2010). Therefore, an overall preference to avoid others’ suffering, relative to one’s own suffering, has selective value, especially when reciprocity is salient. In such situations, those who are more cautious when deciding about others’ pain would thus be less likely to suffer the costs of such punishments. Such preferences could either be innate or learned through social experience. In our study, the experimental design makes a concern for reciprocity salient, as participants decide for each other, so this could make precautionary strategy more likely. In the context of decisions for others involving risk and time, the hypothesis that reciprocity triggers precautionary preferences, which may be moderated by social uncertainty, makes specific predictions.

In time discounting for others, there are two predictions stemming from our proposed model. First prediction is that deciders will be more impatient by choosing *sooner*-smaller payoffs when deciding for others, relative for self, which is exactly what we observed. This is because the proposed (reciprocity → precautionary preference) mechanism should motivate deciders to minimize the potential harm to others, which is the delay until receiving the reward. Note that according to the standard cost–benefit framework in economics (Kreps, 1990), intertemporal choice involves a trade-off between money (benefit) and time/delay (cost), which is why longer delays demand bigger payoffs. Psychologically, it is also likely that in discounting with rewards, in which both outcomes are framed as “gains,” the delay is the most salient cost. Nevertheless, an alternative assumption may be that if decision-makers want to minimize the harm in their decisions for others, then they may choose waiting, because a more salient cost than the cost related to waiting is the cost related to receiving less money (i.e., people might believe that losing money is more harmful than losing time). In support of the delay-as-cost assumption, we conducted a statistical analysis showing that the observed behavioral effect is moderated by participants’ individual discount rates. Specifically, the more impatient deciders should be more likely to treat time as a worse cost. The prediction is that high discounters focus on the cost of delay, and, therefore, have a bigger difference between self and other discounting, in the direction of discounting more for others. We did a median split of the participants into high and low discounters group respectively, and the average difference between the discounting parameters for self and other was significantly bigger in the high discounting group (*M* = 0.118) than the low discounters group (*M* = 0.004), *Z* = 2.46, *p* = .014. Note also that increased impatience in choices for others cannot not driven by increased concavity in the utility function for money for others; because even though the preference elicitation procedure does not correct for curvature in the utility function, this explanation implies the decision-makers should also be more risk averse for gains when deciding for others—in fact, we observed the opposite pattern in the risk-taking task.

Second prediction is that the more uncertain decision-makers are about others, the more precaution they will exercise by choosing *sooner* (smaller) payoffs. Note that social uncertainty stems from individuals not knowing as much about other people’s time preferences and future plans as they do about their own preferences and plans (especially when the recipients are anonymous strangers). This might make decision-makers concerned about choosing delayed rewards for other people because, for example, they do not know if the other person might hate waiting, might have immediate needs, might be leaving the country and closing their bank account, or might otherwise not be able to receive the delayed reward. In line with this intuition, Bartels and Rips (2010) demonstrated that decisions for others tend to be more impatient when a recipient is expected to undergo a significant change in circumstances or a life-changing event. In formal terms, uncertainty about others would amount to less precise estimate of another person’s future states compared with one’s own future states. Therefore, if our participants are uncertain about others’ states, they may also judge providing the other person with *sooner* rewards to be the favorable, “safer” option. Our analysis supported this prediction—more discounting for others when uncertainty parameter is higher for others.

In risky choice for others, the proposed mechanism also makes two key predictions. The first prediction is that we should observe bigger gain–loss asymmetry in risk preferences when deciding for others compared with oneself, that is, more risk-averse choices in the domain of losses, relative to gains, when choosing on behalf of others. This is because precautionary preference commands more risk aversion with others’ money when choosing between losing a fixed amount (e.g., £-3) for sure versus a gamble offering several negative outcomes (e.g., £-1, £-1.25, £-5.75, £-6). In such cases, choosing the fixed £-3 loss avoids the worst potential harm (£-6), which is the most salient cost. This prediction is in line with Paul Slovic’s (1987) research in risk perception, which has identified a number of factors that contribute to perception of risk, including the potential for large or catastrophic losses and affective reactions such as sense of “dread.” More recent research has also demonstrated that the top-ranking risk factors are related to the possibility for large loss of the invested money and the feeling of loss of control over the course of the investment (Vlaev, Chater, & Stewart, 2009). In contrast, when all choice outcomes are gains, there is no potential harm in selecting the gamble, which implies that, given the uncertainty about others’ preferences, decisions for others should be close to risk neutrality, whereas decisions for self will be risk averse as observed before (Rieger, Wang, & Hens, 2015; Vlaev, Stewart, & Chater, 2008). In summary, the proposed processes imply the deciders will be more risk averse for losses and more risk seeking for gains when deciding for others relative to self, which will result, as we observed, in bigger gain–loss asymmetry in risk preferences when deciding for others.^6^

The second prediction is that social uncertainty about others’ preferences will moderate precautionary decision-making under risk. Specifically, precautionary harm aversion predicts avoiding the worst harm in the loss domain, which commands choosing more risk aversely—as all options are losses, the choice between a certain loss and a gamble offering several potential losses (some bigger and some smaller than the certain loss) should make the certain-smaller-loss more attractive (it minimizes the maximum potential harm). In the gain domain, in contrast, the choice is between a certain gain and a gamble offering several gains (some bigger and others smaller), so there is no potential harm when choosing the gamble; therefore, under conditions of uncertainty about others’ needs and wants, one should focus on attaining the maximum possible payoff and be more risk seeking. Therefore, when deciding for others, the valence effect should be larger when deciders are more uncertain; this is exactly what we observed.

### Alternative Explanations

Hsee and Weber (1997) outlined two hypotheses concerning preferences for self and other, which predict uniquely different behavioral choices: *default preference* and *preference-as-feeling*.^7^ The default preference hypothesis, analogous to the false consensus in social psychology (Marks & Miller, 1987), assumes that people use their own preference to predict that of others and, as such, assume others have the same preference as themselves. This hypothesis predicts similar choices for self and other, which was not observed in our data.

The preference-as-feeling hypothesis is based on a dual-process model assumption that one’s preference is an expression of one’s feelings toward each choice option. Dual-process models propose that *reflective* or *experiential* systems differentially dominate choices for self and others. Specifically, when people make choices that go against their goals, such choices often are based on affective evaluations, an account known as the risk-as-feeling hypothesis (Hsee & Weber, 1997; Loewenstein, Weber, Hsee, & Welch, 2001; Stone et al., 2002). This model suggests experiential processes, rewards and punishments, weigh more heavily in decisions for self than for others (Beisswanger et al., 2003; Nicolle, Symmonds, & Dolan, 2011; Rolls, 2005). In decisions about time, contrary to our results, this model predicts more impatient (discounted) choices for self, due to feelings driving the person to choose more immediate payoffs for self (McClure et al., 2004) and difficulty imagining the other person having as strong feelings (Fernandez-Duque & Wifall, 2007; Hsee & Weber, 1997). In decisions about risk, the preference-as-feeling hypothesis predicts more risk-averse choices for self, for both gains and losses, because people would decide for others partly on their own emotions. This often favors risk avoidance (Loewenstein et al., 2001), but due to difficulty considering others as having feelings as strong as one’s own, this preference should regress toward risk neutrality. This prediction does not explain the observed bigger gain–loss asymmetry in risk preferences when deciding for others. In summary, we do not find support for the preference-as-feeling model in intertemporal and risky decisions for self and others in reciprocal setting.

## Conclusions

In the context of decisions for others involving risk and time, our experiments reveal how reciprocity may prompt precautionary preference, which is moderated by social uncertainty. This phenomenon might be more likely to occur when agents, as in our design, do not have information about the others’ particular goals. This is an important topic, because many important relationships involve decisions for each other, such as team situations in which strangers are brought together to form a team with others to perform a task. Such reciprocal decision-making is also found in large organizations where deciders do not personally know the individuals affected by their decisions, but they realize those individuals are also making decisions affecting them. In such situations, the precautionary preferences may be amplified because the beneficiaries are often detached and abstract agents or groups, which brings uncertainty about their preferences and goals. Reciprocal decisions are also found in close relationships such as couples and friendships. In contrast, traditionally, research on surrogate decision-making has focused on one-sided (e.g., economic or medical) decisions taken not by the person who will ultimately gain or lose from the outcome, but instead by someone else (such as policy makers, managers, doctors, carers, next-of-kin, and even strangers in a lab).

Our modeling also revealed that choices are more random when deciding for others for both risk and time. This reveals increased uncertainty in decision-making for others, which is reflected in choice noisiness, or more formally the reliability with which decision values are translated into choices (De Martino et al., 2013). However, an alternative interpretation of the finding, that choices are more random when deciding for others, is in terms of differences in motivation—because people are more deliberate with their own money and less motivated to decide for others. There are two objections against this interpretation of the data. First objection is that the evidence is against such motivational accounts of self–other differences (Kray, 2000). Second objection is that such interpretation does not predict the specific patterns observed in our data. Choosing randomly and caring less about others in the risk task implies that decisions and risk preference parameters should average around risk neutrality for both gains and losses, that is, the valence effect should be smaller for others, which is the opposite of what we found. In the discounting study, more random preferences and decisions predicts more discounting for others, in line with what we found, but random decision-making implies that both the average proportion of delayed choice and the discounting parameter (γ) for others should be around 0.50, not 0.11, as we observed (i.e., the random-choice hypothesis predicts much more extreme levels of discounting than observed in our data).

To corroborate our findings, future research should study the neural mechanisms involved in reciprocal decisions for others, especially when the beneficiary’s preferences for time and risk are not explicitly stated as in our task. Further research should also systematically investigate how concerns about reciprocity influence precautionary preferences (e.g., a condition in which decisions are made for other vs. a condition in which decisions are made for other who also makes decision for self), including also independent measures of individual’s reciprocity and confidence (that the choice is the one that the beneficiary would pick). Another potential research direction would be to build computational models of decision-making, which model the uncertainty in terms of belief distributions about the future for self and other and, thus, tease apart the interaction between uncertainty and precautionary preferences.

In summary, our two experiments reveal unique biases in both the preferences and in the decision rules used to make a choice that satisfies one’s own preferences compared with making choices on behalf of someone else, which is in line with the multistage general theoretical framework employed to understand decision making (DellaVigna, 2009). Those results also demonstrate how computational models can reveal the underlying processes in decision-making. In politics and business, being aware of those decision processes and the self–others discrepancy in choice, may help policy makers and managers introduce policies that better reflect people’s willingness to trade off risk, time, and benefits.

## Figures and Tables

**Table 1 tbl1:** Intertemporal Choice According to Beneficiary (Self and Other)

Measure	Beneficiary				*N*
	Self		Other		
	*M*	*SE*	*M*	*SE*	
Time discounting (γ)	0.07	.02	0.11	.03	41
Choice randomness (β)	2.43	.54	1.68	.49	41
Proportion later choices	0.80	.03	0.75	.03	41

**Table 2 tbl2:** Risky Choice According to Beneficiary (Self vs. Other) and Payoff Valence (Gain vs. Loss)

Measure	Beneficiary	Valence	Statistic		*N*
			*M*	*SE*	
Proportion risky choices	Self	Gain	0.44	.028	40
		Loss	0.40	.027	40
	Other	Gain	0.49	.030	40
		Loss	0.40	.032	40
Risk aversion parameter (ρ)	Self	Gain	−0.04	.033	40
		Loss	−0.07	.017	40
	Other	Gain	0.00	.045	40
		Loss	−0.13	.049	40
Choice randomness parameter (β)	Self	Gain and loss	3.45	.208	40
	Other	Gain and loss	2.61	.284	40

## Footnotes

1 Because payoff probabilities and delays will be explicitly presented in our decision tasks, we will model only changes in “preferences” and “decision rules,” not “beliefs.”

2 Note that this procedure also controls for the effect of confounding factors such as choice fatigue or depletion due to the number of choices in each session. This is because such factors can only lead to randomness and inconsistency in choice, which should lead to worse fit of the model, and we have addressed this issue by excluding from the modeling part such participants.

3 We also analyzed the effect of gender on choices but did not find significant results. The differences between the conditions were examined with a mixed-model two-way (Beneficiary × Gender) univariate analysis of variance (general linear model), with beneficiary as within-subject variable, participant gender as between-subjects variable, and the mean proportion of choices of the *later* (delayed) option as the dependent measure. There was a significant main effect of beneficiary, *F*(2, 86) = 6.93, *p* = .002 (η^2^ = .14). Post hoc analysis revealed that after Bonferroni adjustment for multiple comparisons, the proportion of *later* picks for self was significantly higher than the proportions for both male (*p* = .010) and female (*p* = .043) others, and male was not significantly different from female others (*p* = 1.000). There was no significant main effect of the participant’s own gender, *F*(1, 43) = 0.54, *p* = .467 (η^2^ = .01), suggesting that male and female participants did not exhibit different levels of impatience (i.e., delay discounting). The Beneficiary × Gender interaction was also not significant, *F*(2, 86) = 0.30, *p* = .739 (η^2^ = .01), which means that male and female participants did not respond differently when choosing for a same- or opposite-sex beneficiary (i.e., the degree of time discounting, indicated by the proportions of *later* choices, did not vary depending on the gender of the decision maker and the beneficiary, respectively).

4 We did not fit any version of prospect theory because the mean-variance model has been consistently shown to outperform prospect theory in this task (Wright et al., 2012; Wright, Symmonds, Morris, & Dolan, 2013).

5 Similarly to Wright et al. (2012), we also explored interindividual differences to seek further evidence of behavioral independence between the influences of risk and valence. If these influences result from stable and independent processes, we can predict that knowing an individual’s sensitivity to one influence would not predict sensitivity to the other. We conducted additional analysis, in which for each participant, the average proportion of riskier choices indexes risk preference and the difference in riskier choices between gain and loss domains indexes the impact of valence. Crucially, these preferences were independent, with risk and valence effects showing no correlation across subjects for either self (*r* = .03; *p* = .928) or other (*r* = −0.159; *p* = .270).

6 This asymmetry in preferences depending on the gain/loss domain is also known and the “valence effect,” which is not, however, equivalent to the “framing effect” in the behavioral economics literature. The framing effects exist when the same information is provided in both conditions, just framed differently, which usually leads to “loss aversion”—more risk seeking in the loss frame (see De Martino, Kumaran, Seymour, & Dolan, 2006). In our task, the loss situation and the gain situation are different. Thus, contrary to the framing effect, being risk averse in one situation and risk seeking in another is not inherently “irrational” in our task.

7 Hsee and Weber (1997) only examined whether people can accurately predict the risk preferences of others (i.e., they did not study time preferences and did not ask the participants to actually make decisions on behalf of others).

## A Experimental Instruction for Experiment 1: Intertemporal Choice for Self and Others

Welcome to the experiment! Take time to read these instructions carefully. If you do have any questions, please raise your hand and one of the coordinators will come to you and answer it privately. Please do not talk to anyone during the experiment.

In the paragraphs below, we will describe the experiment. First, as this experiment differs from most others in the lab, we need to provide some explanation about how the payments will work. In this experiment, in contrast to others run in the lab:

1 You will NOT be paid in cash. We will be paying using electronic payments. Either we can credit the money into a PayPal account or directly into your U.K. bank account; you will need to provide details of one of these accounts.
2 Payments will not necessarily be made on the day of the experiment (this depends on choices made during the experiment). Some payments may be made *later* on (but even those paid on the day of the experiment will be paid electronically).
3 There will not be a show-up fee. The show-up fee element will be replaced by higher payments possible from decisions made during the experiment. Payments will be on average higher than most experiments of similar duration.

If you have any queries about this, please raise your hand and the experimenter will come and answer it privately.

Now, about the experiment!

In this experiment, you will make 240 choices about different amounts of money to be paid. Specifically, each choice will have the following format:

“Choose £X1 in Y1 weeks OR £X2 in Y2 weeks” where ×1, ×2, Y1, and Y2 are numbers. If Y1 or Y2 are equal to zero, that means “today.” You will make your choice by clicking on one of the two buttons on the screen.

Not all 240 choices are made on behalf of yourself, nor will you be paid for every choice. In fact, one third of the choices relate to payments to you, one third to another subject in the laboratory that is male and to another subject in the laboratory that is female.

The choices where you are deciding for yourself will have a screen with a black surround, the choices where you are deciding for another subject (male) in the laboratory will have a light blue surround, and the choices where you are choosing on behalf of another subject (female) in the laboratory will have a pink surround. The 240 choices will be in six blocks of 40 choices where each of the 40 choices has the same beneficiary.

After you have made your 240 choices, the computer will choose ONE choice at random to be paid. If the payment choice is a decision where you chose on behalf of someone else, then they will get the payment you chose, AND you will get the payment they chose (although not necessarily for the same decision problem).

At the end of the experiment, there will be a short survey and then we will arrange the payment information and provide receipts.

All data from this experiment and questionnaire are held anonymously. That is, there is no unique link that could identify you with the data.

## B Experimental Instruction for Experiment 2: Risky Choice for Self and Others

Thank you for taking part in the experiment today. How much you earn depends on the choices made in the task, as well as an element of chance.

During this experiment, *half the decisions you make will be on behalf of someone else* and *half on behalf of yourself.* The person you decide on behalf of today (*your partner*) will also be deciding on your behalf. In the experiment, each participant will receive a starting amount of £12, will then gain or lose money from the decisions made on their behalf by their partner, and will also gain or lose money from their decisions made on their own behalf. The whole experiment takes about 25 min, of which the first half is for one beneficiary (your partner or yourself) and the second half for the other beneficiary. You will also receive £5 for attending (the show-up fee).

### What Happens in Each Trial?

The structure of each trial is the same, whoever the beneficiary (your partner or yourself). In each trial, you have to make a choice between a *gamble option* and a *sure option*. Sometimes the decisions will be about gaining money (the *gain trials*) and sometimes about losing money (the *loss trials*). If you choose the sure option the beneficiary will get the outcome shown for certain, which is always gaining £3 for certain in the “gain trials” and losing £3 for certain in the “loss trials.” If you pick the gamble option, the beneficiary will get one of the outcomes shown on the pie chart, with the chance of getting that outcome shown by the size of that segment.

In contrast to most other experiments you may have encountered at ELSE, you do not have a long time to think about your decision. What happens on each trial is as follows:

1 The trial decision will appear on the screen (see example screenshots below).
2 After about 4 s to think about your choice, decision buttons will appear on the screen (two buttons marked “GAMBLE” and “SURE”).
3 You have about 2 s to click one of the buttons to register your choice, after which the experiment will move onto the next trial. It is always better to make a choice within the 2 s, as if you don’t make a choice in the “gain trials” the beneficiary will get zero and in the “loss trials” the beneficiary will lose £6.

You will not receive any feedback during the experiment. Instead, at the end, *one of the gain trials* and *one of the loss trials* that *you decided about on behalf of your partner* and also one *gain trial* and one *loss trial* that *you decided about for yourself* will be selected at random and played out for real.

So, at the end of the experiment, you will see four trials that you made decisions in, and they will be played out. Your earnings and partner’s earnings for you will be added to the £12. After that, there will be a summary of the results.
